# Antidepressant effects of ketamine: mechanisms underlying fast-acting novel antidepressants

**DOI:** 10.3389/fphar.2013.00161

**Published:** 2013-12-27

**Authors:** Caroline A. Browne, Irwin Lucki

**Affiliations:** ^1^Department of Psychiatry, University of PennsylvaniaPhiladelphia, PA, USA; ^2^Department of Pharmacology, University of PennsylvaniaPhiladelphia, PA, USA

**Keywords:** ketamine, antidepressants, depression, animal models, BDNF

## Abstract

Newer antidepressants are needed for the many individuals with major depressive disorder (MDD) that do not respond adequately to treatment and because of a delay of weeks before the emergence of therapeutic effects. Recent evidence from clinical trials shows that the NMDA antagonist ketamine is a revolutionary novel antidepressant because it acts rapidly and is effective for treatment-resistant patients. A single infusion of ketamine alleviates depressive symptoms in treatment-resistant depressed patients within hours and these effects may be sustained for up to 2 weeks. Although the discovery of ketamine's effects has reshaped drug discovery for antidepressants, the psychotomimetic properties of this compound limit the use of this therapy to the most severely ill patients. In order to develop additional antidepressants like ketamine, adequate preclinical behavioral screening paradigms for fast-acting antidepressants need to be established and used to identify the underlying neural mechanisms. This review examines the preclinical literature attempting to model the antidepressant-like effects of ketamine. Acute administration of ketamine has produced effects in behavioral screens for antidepressants like the forced swim test, novelty suppression of feeding and in rodent models for depression. Protracted behavioral effects of ketamine have been reported to appear after a single treatment that last for days. This temporal pattern is similar to its clinical effects and may serve as a new animal paradigm for rapid antidepressant effects in humans. In addition, protracted changes in molecules mediating synaptic plasticity have been implicated in mediating the antidepressant-like behavioral effects of ketamine. Current preclinical studies are examining compounds with more specific pharmacological effects at glutamate receptors and synapses in order to develop additional rapidly acting antidepressants without the hallucinogenic side effects or abuse potential of ketamine.

## Introduction

Major depressive disorder (MDD) is a serious public health problem and one of the most common psychiatric disorders, with a lifetime prevalence of 17% in the United States (Kessler et al., 2005). Although the currently available antidepressants provide a measurable degree of therapy, approximately 50% of individuals diagnosed with MDD do not respond adequately to first–line treatment with conventional antidepressants (Trivedi et al., 2006; Fava et al., 2008). Moreover, the 3–4 week delay in the onset of therapeutic efficacy is particularly difficult for patients with persistent suicidal ideation. Patients that emerge as treatment resistant, defined as failing two or more trials of medication, are more severely ill with comorbid anxiety disorders and are at increased risk of suicide for an extended period of time (Joffe et al., 1993; Souery et al., 2007; Schosser et al., 2012). Therefore, there is a pressing medical need to develop rapidly acting therapeutics that are capable of immediately relieving the depressive symptomology, and persisting in their action as an antidepressant, for patients unable to respond to conventional therapies.

Recently it has been demonstrated that the NMDA receptor antagonist ketamine has rapid-acting and transient antidepressant effects in patients that are treatment resistant (Mathew et al., 2012). However, the discovery of ketamine is no panacea. The psychotomimetic properties and abuse potential of ketamine necessitate caution in promoting this particular compound as a general treatment for MDD. Understanding the underlying mechanism of action of ketamine linked to behavioral improvement is of significant importance for the development of novel, more improved antidepressants beyond the use of ketamine. This review will focus on the molecular alterations and animal behavior studies that have been used to measure potential correlates of the antidepressant effects of ketamine. As ketamine produces clinical antidepressant effects with a different time course and apparently different neurochemical mechanism than conventional antidepressants, the results of these studies have revealed new paradigms that can be used to identify novel compounds which may have a similar therapeutic potential and time course as ketamine in targeting treatment resistant depression (TRD).

## Ketamine—clinical trials

The initial clinical trials were double blind crossover studies that utilized a single infusion of ketamine (0.5 mg/kg) administered intravenously over a 40 min period (Berman et al., 2000; Zarate et al., 2006). Berman et al reported decreases in depressive symptomology, which emerged progressively over the first 3 days in all of the eight patients that were treated; one patient continued to show antidepressant-like effects 2 weeks post-infusion. Similarly, Zarate and colleagues reported a significant and rapid alleviation of depressive symptoms in 12 individuals on the first day, with six subjects exhibiting symptom alleviation for a least 1 week; two of these subjects continued to show antidepressant effects 2 weeks post-single ketamine infusion. Subsequent studies reported significant efficacy of ketamine in reducing suicidal ideation in individuals exhibiting TRD (Diazgranados et al., 2010). Moreover, a proof of concept trial conducted in treatment-resistant bipolar patients revealed a more rapid onset of antidepressant effects following ketamine infusion concomitant to their valproate and lithium treatment compared to previous studies conducted in MDD patients. However, the alleviation of depressive symptoms in the bipolar study persisted for only 3 days compared to the 7 days reported in earlier trials. In addition, ketamine had significant efficacy in patients resistant to electroconvulsive therapy (ECT) and produced more rapid antidepressant effects compared to ECT (Ibrahim et al., 2011). Unlike the almost immediate alleviation of depressive symptomology associated with ketamine infusion, similar reductions in symptoms were observed approximately 1–2 weeks following the first of the thrice-weekly ECT exposures. Furthermore, the use of ketamine as the anesthetic prior to ECT has been suggested to improve outcome and response to ECT (Hoyer et al., 2013). Indeed, the administration of ketamine/propofol (ketofol) improved the severity of seizure duration, induced an earlier onset of the antidepressant effect and significantly improved cognitive performances compared to propofol (Wang et al., 2012). Recently, it was reported that subanesthetic doses of S-ketamine with propofol actually worsened the post-treatment disorientation in some patients (Jarventausta et al., 2013). Further research is ongoing to determine the benefit of the S-enantiomer over the commonly used racemic mixture of ketamine. One group suggested that S-ketamine did not induce the transient psychotomimetic effects evident in the initial phase of infusion (Segmiller et al., 2013).

An extensive clinical trial involving 67 patients at two sites with documented TRD established the most definitive antidepressant efficacy of ketamine, in comparison with the benzodiazepine, midazolam, used as an active placebo control (Murrough et al., 2013). The response rates to ketamine vs. midazolam were 64 and 28%, respectively, with ketamine significantly reducing scores in the MADRS by 7.95 points. Ketamine-treated patients continued to exhibit improved scores over the 7-day period post-infusion compared to midazolam, however, the reduction of depressive scores on day 7 was no longer significant. Although most studies of ketamine have involved only a small number of patients, this is the best-designed and most extensive clinical trial to confirm the efficacy of ketamine in rapidly and persistently alleviating depressive symptomology.

Because the clinical effects of ketamine are transient, studies have assessed the efficacy of ketamine administration when given chronically. Significant improvement of symptoms persisted following six infusions of ketamine over 11 days, although the 9 patients treated in this trial eventually relapsed 19 days after the final infusion (aan het Rot et al., 2010). In addition, the effects of oral administration of ketamine given over a long-term period yielded positive findings, with patients exhibiting improved mood over the 28-day treatment period. Interestingly, although the level of symptom alleviation was the same as that achieved by I.V. infusion of ketamine, oral ketamine did not elicit a significant effect on depressive symptoms until day 14 of treatment but fortunately did relieve anxiety symptoms within 3 days of treatment (Irwin et al., 2013). Psychotomimetic effects were not observed in these patients; however, there were some reports of sleep disturbances and diarrhea. Moreover, another study conducted in bipolar patients using sublingual ketamine indicated significant (70%) numbers of individuals exhibiting improved mood with limited side effects with rapid onset of action. These data indicate that further evaluation of the administration route of ketamine and their side effect profiles may be beneficial.

Although there is a clear consensus on the rapidity of the antidepressant effect of ketamine in TRD, with most patients experiencing elevated mood starting approximately 120 min post-infusion, not all patients respond to ketamine treatment. Response rates across studies have ranged between 25 and 85% at 24 h and 14–70% at 72 h (Aan Het Rot et al., 2012). In addition, the duration of the antidepressant effect has varied across studies. In most of the trials conducted so far, only approximately half of the patients exhibited relief of depressive symptoms from ketamine lasting past 72 h. The reasons underlying variability in the response to ketamine are unknown. Given the heterogeneous nature of depression, a number of genetic, environmental and patient characteristics may be associated with treatment response. For example, patients with a family history of alcohol use disorder (AUD) exhibit better outcomes in response to ketamine administration, reporting less psychotomimetic disturbances and greater reductions of depression symptoms, compared to MDD patients without a history of AUD (Phelps et al., 2009). In addition, potential biomarkers or genetic variants will likely be found to augment or prevent responsiveness to ketamine.

Some clinical studies have tried to identify the critical pharmacological characteristics of ketamine associated with treatment response. Modification of the NMDA receptor subunit NR2B may confer an increased treatment response; indeed, NR2B antagonists, CP-1016060 and MK-0657 have shown good efficacy in treating TRD patients (Preskorn et al., 2008; Ibrahim et al., 2012a). AZD6765, a NMDA channel blocker, was assessed for its antidepressant-like qualities in a double blind crossover study involving 22 subjects. Although no psychotomimetic effects of this compound were reported, depressive symptoms were alleviated only for the first 2 h following infusion (Zarate et al., 2013). Similarly, administration of riluzole, (a sodium channel blocker, which indirectly inhibits glutamate release) for 4 weeks following ketamine infusion did not potentiate symptom improvement compared to placebo (Ibrahim et al., 2012b). These reports and a growing literature indicate that the mechanisms of action mediating ketamine's antidepressant effects have not yet been identified and are not elicited simply by the blockade of NMDA receptors.

## Antidepressant-like behavioral effects of ketamine in rodents

The ability of ketamine to affect depressive-like behavior in a number of preclinical behavioral paradigms and models of depression has been widely studied in the past few years. Many reports indicate that acute administration of ketamine produces antidepressant-like effects in rodents (Table 1). However, some of the findings have not been replicated consistently by other laboratories. The literature concerning the antidepressant-like effects of ketamine is reviewed here, focusing on the effects of varying test conditions on behavioral outcomes. In addition, many studies have now reported that the effects of a single dose of ketamine can be measured over a protracted period of time lasting between days to weeks (Table 2). The time course of these protracted effects resembles the time course for ketamine's clinical effects (Yilmaz et al., 2002; Maeng et al., 2008), and may represent a new animal behavioral paradigm that correlates with the clinical effects of rapidly acting antidepressants.

**Table 1:** **Acute effects of ketamine**.

**References**	**Species and strain**	**Ketamine—supplier and dose**	**Behavioral alterations**	**Molecular alterations**
**ACUTE EFFECTS OF KETAMINE**				
Burgdorf et al., 2013	Male adult (2–3 months) Sprague-Dawley rats	Fort Dodge (Butler, USA), I.V., I.P., and S.C. 10 mg/kg	Reduced immobility in FST 20–60 min and 24 h post i.p. Injection (10 mg/kg). Reduced latency to feed in the NIH 1 h post 10 mg/kg i.v.	Increased NR2B and GluR1 expression in the mPFC and HC 24 h post-injection
Carrier and Kabbaj, 2013	Male (250–270 g) and female (200–225 g) Sprague-Dawley rats	Fort Dodge (Butler Schein), Inc. 2.5–0 mg/kg	Latency to feed was reduced in the NSF 24 h post-injection (5 and 10 mg/kg). Increased sucrose consumption of males 48 h post-injection in the SPT. Reduced immobility in FST in males & females 30 min post-injection	Increased mTOR phosphorylation in males and females, reduced eEF2 phosphorylation in males (5 mg/kg)
Gigliucci et al., 2013	Male (280–320 g) Sprague- Dawley rats	Vetoquinol Ltd., UK (1.0 mg/ml). 10–25 mg/kg i.p.	Rats exhibited antidepressant-like effects in the FST at 1 or 24 h after a single injection of ketamine. Ketamine was ineffective following 3 injections (24, 5 and 1 h prior to testing). Ketamine (25 mg/kg) reversed stress-induced immobility; this was prevented by *p*CPA treatment at 24 h but not at 1 h post-injection	Depletion of cortical serotonin levels by *p*CPA (1.0 mg/kg once daily for 3 days) attenuated the antidepressant-like effect of ketamine in the FST
Koike et al., 2013a	Male Sprague-Dawley rats (185–325 g at testing)	Ketalar® Sankyo Yell Pharmaceutical Co., Ltd., 1–10 mg/kg i.p.	Ketamine (10 mg/kg) decreased immobility 30 min post-treatment in rats exposed to 21 days of corticosterone administration	N/A
Koike et al., 2013b	Male ICR (5 weeks) and male C57BL/6j (9 weeks)	Ketalar® Sankyo Yell Pharmaceutical Co., Ltd. 30 mg/kg i.p.	Ketamine decreased immobility in the FST & latency to feed in the NSF at 30 min and 24 h post-injection. K252a prevented ketamine's effects at 24 h.	N/A
Muller et al., 2013	Male Sprague Dawley rats (330–400 g)	Fort-Dodge (Pfizer CT), USA. 15 mg/kg (i.p.)	Reduced immobility in FST 2 h post-injection	Increased p-αCamKII and decreased SNARE complex expression 1– 4 h post-injection. No effect on GSK-3 activity. Protracted increased in synapsin expression1 h to 7 days post-injection
Walker et al., 2013	CD-1 mice (6 wks. old) and C57BL/6J mice (12 weeks old)	Fort Dodge Animal Health 6 mg/kg (i.p.)	Ketamine co-administered with LPS but not pretreatment 24 h prior blocked LPS-induced immobility in FST and anhedonia in the SPT. 10 h post LPS, ketamine administration reversed the anhedonia in SPT, this was blocked by NBQX	Ketamine did not block the LPS-induced increases in kynurenine metabolites, cytokines or BDNF expression at 6–28 h
Iijima et al., 2012	C57Bl/6J mice (9 weeks)	Sigma-Aldrich 30 mg/kg (i.p.)	Latency to feed in the NSF was reduced at 30 min and 24 h post-injection. Rapamycin reversed the 24 h reduction in NSF latency	N/A
Liu et al., 2012	BDNF knockin mice, (Val66Met SNP) Val/Met, Met/Met and Val/Val (WT) 6–8 months	Hospira Inc. 10 mg/kg (i.p.)	24 h post-injection the AD effects of ketamine in the FST were blocked in Met/Met mice	Met/Met knockin mice are insensitive to the molecular effects of ketamine on spine head diameter and spine length modulated in WT mice
Yang et al., 2012	Male Wistar rats (180–220 g)	Gutian Pharmaceutical CO. Ltd., Fuijan, China 10 mg/kg (i.p.)	Reduced immobility in FST 30 min post-injection	Increased mTOR phosphorylation in HC and PFC
Yang et al., 2013b	Male Wistar rats (200–300 g)	Gutian Pharmaceutical CO. Ltd., Fuijan, China 5–15 mg/kg (i.p.)	Dose-dependent reduction in immobility in the FST 30 min post-injection	Increased BDNF levels in the HC following 10 and 15 mg/kg. Dose dependent increase in phosphorylated mTOR levels in HC
Wang et al., 2011	Male Wistar rats (60 days old)	Sigma-Aldrich 15 mg/kg (i.p.)	Decreased immobility in the FST 60 min post-injection	Increased BDNF expression and decreased phosphorylation of GluR1 (Ser845) in HC 60 min post-injection
Beurel et al., 2011	WT and GSK-3 Knock in mice	10 mg/kg (i.p.)	AD effects in LH in WT but not GSK-3 knock-in mice	Increased pGSK-3β (CTX and HC) 30 and 60 min post-injection
Koike et al., 2011a	Male ICR mice (25–35 g)	Sigma-Aldrich 3–30 mg/kg (i.p.)	Ketamine reduced immobility in the TST 24 h post 30 mg/kg injection. Rapamycin reversed the ketamine-induced reduction in TST immobility	N/A
Reus et al., 2011	Male Wistar rats (60 days old)	Fort Dodge Animal Health—0.1 g/ml injectable solution, 5–10 mg/kg	Immobility in the FST was reduced at 60 min postinjection by 10 mg/kg only	Ketamine 5 mg/kg increased the expression of BDNF, CREB, and PKC phosphorylation in the PFC. 5mg/kg increased BDNF in the HC and Amg. 10 mg/kg decreased BDNF in the PFC, HC, and Amg. 10 mg/kg increased CREB expression and PKC phosphorylation in the PFC
Li et al., 2010	Male Sprague Dawley rats (150–250 g)	Sigma-Aldrich 10 mg/kg (i.p.)	Ketamine produced AD effects in the FST, LH and NSF test 24 h post-injection, blocked by rapamycin	Ketamine 10 mg/kg activated mTOR, ERK, and PKB/Akt signaling, blocked by NBQX, Ketamine 10 mg/kg increased expression of certain synaptic proteins at 2, 6, and 72 h post-injection, blocked by rapamycin
Ghasemi et al., 2010	Male NMRI mice (23–30 g)	Sigma-Aldrich 0.5–5 mg/kg (i.p)	Ketamine reduced immobility in the FST 45 min post-injection (2 and 5 mg/kg)	N/A
Cruz et al., 2009	Male Swiss mice (25–35 g)	Sigma-Aldrich 6.35–50 mg/kg (i.p.)	12.5, 25, and 50 mg/kg ketamine reduced immobility in the FST 30 min post-injection. Only 50 mg/kg ketamine reduced immobility in the TST	N/A
Engin et al., 2009	Male Sprague-Dawley rats (180–360 g)	10–50 mg/kg (i.p.)	Ketamine (50 mg/kg) increased the % of open arm entries in the EPM. Both doses decreased immobility in the FST 30 min post-injection	N/A
Rezin et al., 2009	Male Wistar rats (300 g)	Fort Dodge Animal Health 15 mg/kg (i.p.)	Ketamine did not reverse the CMS-induced reduction in consumption of sweet food	Ketamine reversed the CMS-induced reductions in mitochondrial respiratory chain enzymes
Garcia et al., 2008a	Male Wistar rats (60 days old)	Fort Dodge (Brazil) 5, 10, and 15 mg/kg (i.p.)	1 h post-injection ketamine (5 & 10 mg/kg) significantly reduced immobility in the FST	BDNF increased in the HC following ketamine injection (15 mg/kg)
Hayase et al., 2006	Male ICR mice (60–90 days old)	Sankyo Co., Ltd. Tokyo, Japan 30–1.0 mg/kg (i.p.)	Ketamine increased the latency to immobility in the FST and was anxiolytic in the EPM at both doses 60 and 120 min post-injection	N/A
Rosa et al., 2003	Swiss mice male and female (30–40 g)	Sigma-Aldrich 5 mg/kg (i.p.)	Ketamine reduced immobility in the TST 30 min post-injection	N/A
Mantovani et al., 2003	Male Swiss mice 35–45 g)	0.1 mg/kg (i.p.)	Ketamine reduced immobility in the TST 30 min post-injection	N/A

This table outlines studies that have assessed the antidepressant-like effects of ketamine at 30 min to 24 h post-administration in commonly used behavioral tests. Molecular alterations of relevance to ketamine's molecular mechanism of action are also reported. FST, forced swim test; TST, tail suspension test; LH, learned helplessness; NSF, novelty suppressed feeding; SPT, sucrose preference test; EPM, elevated plus maze; AD, antidepressant; CMS, chronic mild stress; LPS, lipopolysaccharide; HC, hippocampus; CTX, cortex; Amg, amygdala; mPFC, medial prefrontal cortex; WT, wild type.

**Table 2:** **Protracted effects of ketamine**.

**References**	**Species and strain**	**Ketamine—supplier and dose**	**Behavioral alterations**	**Molecular alterations**
**PROTRACTED EFFECTS OF KETAMINE**				
Akinfiresoye and Tizabi, 2013	Male WKY rats	Fort-Dodge (Henry Schien), 0.25 and 0.5 mg/kg (i.p.), administered daily for 10 days	Only chronic administration of 0.5 mg/kg reduced immobility in the FST and increased sucrose intake in the SPT	0.25 mg/kg ketamine did not alter mTOR phosphorylation or synapsin 1 and BDNF expression
Liu et al., 2013	Male Sprague-Dawley rats (150–250 g)	Hospira Inc., 1 and 10 mg/kg (i.p.)	Ketamine reduced immobility in the FST 24 h and 1 week following a single 10 mg/kg injection. This effect was not observed 2 weeks post-injection	Ketamine increased p- S6K, p-ERK, p-Akt but not p-mTOR or GSK-3b 1 h post-injection (10 mg/kg). These changes were not detected 24 h post-injection. 5-HT and hypocretin induced EPSCs were increased 24 h following ketamine treatment (10 mg/kg). Ketamine 1 and 10 mg/kg increased spine head diameter and spine density
Ma et al., 2013	C57Bl/6J mice (7 wks. old 20 g)	Gutian Pharmaceutical CO. Ltd., Fuijan, China. 10 mg/kg (i.p.)	Ketamine reversed CMS-induced increases in immobility in the FST and TST 48 h post-treatment. Ketamine reversed CMS-induced reductions in sucrose intake in the SPT, 24 h, 4, 6, and 8 days post-treatment. In non-stressed animals ketamine reduced immobility in the TST and FST at 3 and 24 h post-injection	N/A
Parise et al., 2013	Male adolescent Sprague-Dawley rats (post-natal day 35–49)	Fort-Dodge (Schein), 5, 10, and 20 mg/kg (i.p.). Administered twice a day for either 1 or 15 days	Ketamine (10 and 20 mg/kg) reduced immobility in the FST 24 h after the 2nd injection. CMS-induced immobility was reversed by ketamine (20 mg/kg). No effect of ketamine on SPT was observed. Two months after chronic ketamine treatment rats exhibited an anxiolytic phenotype on the EPM and AD effects in the FST	N/A
Lindholm et al., 2012	Adult male C57Bl/6J and WT & BDNF ± mice	Sigma-Aldrich 20 and 50 mg/kg (i.p.)	Decreased immobility in FST in WT mice at 45 min but not 7 days post-injection	No alterations in TrkB phosphorylation at 60 min or 7 days post-injection
Tizabi et al., 2012	Male and Female WKY and Wistar rats	Fort-Dodge (Schein), 0.25–5 mg/kg (i.p.), administered once or daily for 10 days	No acute/chronic effect of ketamine on Wistar immobility levels in the FST. 2.5 and 5 mg/kg reduced immobility of WKY rats in the FST; the 5 mg/kg dose had protracted effects 1 week post-injection. Chronic administration of 2.5 and 5 mg/kg reduced immobility of WKY but not Wistar. The effect of the 2.5 mg.kg dose were evident 1 week following the cessation of treatment	Ketamine (chronic 0.5 mg/kg paradigm) increased AMPA receptor binding & the AMPA/NMDA ration in WKY rats
Autry et al., 2011	Adult male C57BL/6 WT and inducible BDNF KO mutants	Fort Dodge Animal Health 3 mg/kg (i.p.)	No effect in EPM or fear conditioning 24 h post-injection. Reduced FST immobility at 30 min, 3 h, 24 h, and 1 week, blocked by NBQX. Reduced latency to feed in NSF, increased sucrose intake & decreased immobility in CMS mice 30 min post-injection. Rapamycin did not block ketamine-induced reductions in FST immobility 30 min post-injection. Anisomycin prevented the effects of ketamine in the NSF & FST. TrkB KO mice did not response to ketamine	Increased TrkB activation. Increased BDNF protein but not mRNA at 30 min and 1 h post-injection. Decreased phosphorylation of eEF2 in HC. Blocked spontaneous activity of NMDARs in HC cultures
Bechtholt-Gompf et al., 2011	CD-1 and BALB/c mice	Sigma-Aldrich, dose range 0.5–3.0 mg/kg	Reduced immobility in TST 1 h post-injection (1.0 mg/kg), not observed at day 7. No effect on FST immobility at any dose, or time point	N/A
Koike et al., 2011b	Male ICR mice (25–35 g) and male Sprague-Dawley rats (230–350 g)	Sigma-Aldrich 3–30 mg/kg (i.p.)	Ketamine reduced the number of failures to escape in the LH test 30 min post 10 mg/kg injection. Reduced immobility in the TST 30 min & 72 h post 30 mg/kg injection	N/A
Li et al., 2011	Male Sprague Dawley rats (150–250 g)	Sigma-Aldrich 10 mg/kg (i.p.)	Ketamine reversed CMS-induced anhedonia in the NSF test 2 days post-injection. Sucrose consumption was increased 1, 3, 5, and 7 days following the single ketamine injection	Ketamine reversed CMS-induced deficits in synaptic EPSCs, spine density and synaptic protein expression. At 7 days post-treatment these effects were still apparent
Yilmaz et al., 2002)	Male Wistar rats (280–310 g)	Parke-Davis 50 mg/ml stock 1.0 mg/kg (i.p.)	Ketamine reduced FST at 3, 7, and 10 days post-injection, (this was only in the second test of each day).	N/A
Garcia et al., 2009	Wistar rats (300–350 g)	Fort Dodge Animal Health 15 mg/kg once on day 7 or daily for 7 days	CMS-induced reductions in sucrose intake, weight loss, adrenal hypertrophy, and increased ACTH and corticosterone levels were reversed by acute and chronic ketamine administration. Chronic ketamine increased sucrose intake in controls	No differences in HC BDNF concentrations
Garcia et al., 2008b	Wistar rats (300–350 g)	Fort Dodge Animal Health, 5, 10, and 15 mg/kg—daily i.p. injections for 12 days	All doses reduced immobility in the FST	HC BDNF concentrations were not altered
Popik et al., 2008	Male Wistar rats (270 g) and male Sprague Dawley rats (275 g), C57/Bl/Han male mice (24 g) and Male Swiss mice (28 g)	Biowet, Pulawy, Poland, FST rats, 1.0 mg/kg. TST mice, 50–1.0 mg/kg. FST mice, 1.25–10 mg/kg	Reduction of immobility in the FST in mice but not in rats at 30 min post-injection only (50 mg/kg). Ketamine reduced immobility in the TST at 40 min but not at 1 week post-injection	N/A
Maeng et al., 2008	Mice	Sigma-Aldrich 0.5–10 mg/kg (i.p.)	Ketamine reduced the number of escape failures in LH 24 h post-injection. Ketamine (2.5 mg/kg) reduced immobility in the FST at 30 min and 2 weeks post-injection	Ketamine reduced phosphorylation of HC GluR1 (S845), rescued by NBQX

This table outlines studies that have assessed the antidepressant-like effects of ketamine from day 2, or, 48 h post-administration onwards in commonly used behavioral tests. In some of these studies earlier time points have been assessed, the results are also included in this table. Molecular alterations of relevance to ketamine's molecular mechanism of action are also reported. FST, forced swim test; TST, tail suspension test; LH, learned helplessness; NSF, novelty suppressed feeding; SPT, sucrose preference test; EPM, elevated plus maze; AD, antidepressant; CMS, chronic mild stress; LPS, lipopolysaccharide; HC, hippocampus; CTX, cortex; Amg, amygdala; mPFC, medial prefrontal cortex; WTm, wild type.

### Forced swim test (FST)

The FST is the most frequently used behavioral test for measuring depressive-like behavior in rodents. It has also been a frequently used test within the preclinical ketamine literature. Mice and rats placed in cylinders containing water rapidly become immobile, demonstrated by floating passively or making only movements necessary to remain afloat. Based on an immobility response induced by inescapable exposure to stress, the FST also has strong predictive validity because short-term administration of antidepressant compounds from a variety of pharmacological classes reduces immobility time in the FST. These drugs include tricyclic compounds, MAO inhibitors, atypical antidepressants, and SSRIs (Cryan et al., 2005). Furthermore, the behavioral effects of tricyclics and SSRIs do not last beyond a few hours following their acute administration (Hoshaw et al., 2008).

Several groups have reported that a single administration of ketamine produced acute reductions of immobility in the FST shortly after injection (Table 1). Although the majority of these studies utilized a 10 mg/kg dose administered intraperitoneally (i.p.), subanesthetic doses of ketamine ranging from 10–50 mg/kg have produced antidepressant-like effects in the FST. However, some studies failed to detect acute effects of ketamine using the FST in mice (Bechtholt-Gompf et al., 2011) or in rats (Popik et al., 2008).

A feature of ketamine's pharmacology distinct from conventional antidepressants is that it produces protracted behavioral effects persisting between one to several days after administration (Table 2). The majority of studies indicate that the FST remains sensitive to the protracted effects of ketamine up to 1 week after a single injection (Table 1). These protracted effects were reported to persist for 8 days (Ma et al., 2013), 10 days (Yilmaz et al., 2002), 12 days (Garcia et al., 2008a), and 2 weeks (Maeng et al., 2008). Interestingly, antidepressant-like effects of ketamine were observed in the FST 2 months following the cessation of a 15-day treatment of rats during adolescence (Parise et al., 2013). This result is in line with other studies that have used a 10 or 12-day dosing regimen to establish longer-lasting effects of chronic ketamine on depressive-like activity in the FST (Tizabi et al., 2012; Akinfiresoye and Tizabi, 2013). Only one study examining the protracted effects of ketamine failed to report this finding (Lindholm et al., 2012).

The presence of chronic stress has been shown to facilitate the detection of antidepressant-like effects of ketamine in the FST (Koike et al., 2013a). There are also significant strain differences in the sensitivity to ketamine. For example, Wistar rats are insensitive to the antidepressant-like effects of low dose ketamine (2.5 and 5 mg/kg) following chronic treatment. In contrast, WKY rats were extremely sensitive to ketamine-induced reductions in FST immobility (Tizabi et al., 2012). WKY rats have a high baseline immobility level in the FST, which may allow for a greater sensitivity to compounds. Moreover, WKY rats are a genetic model of pathological depression and anxiety (Will et al., 2003; Solberg et al., 2004), which could provide them greater sensitivity to the effects of ketamine. Finally, the WKY strain is insensitive to SSRIs (Lopez-Rubalcava and Lucki, 2000; Tejani-Butt et al., 2003; Will et al., 2003) showing that ketamine is active under conditions where current antidepressants are ineffective. This feature makes WKY rats a useful strain in which to assess novel compounds resembling ketamine, which may be screened for efficacy in TRD.

### Tail suspension test (TST)

The TST is widely used in the preclinical ketamine literature as a less stressful test of behavioral despair when mice are suspended from their tail (Steru et al., 1985; Cryan et al., 2005). TST has predictive validity because it measures antidepressant-like responses from various classes of drugs. Ketamine reduces immobility levels in mice acutely, with studies reporting reductions in immobility time at 30 min (Mantovani et al., 2003; Rosa et al., 2003; Cruz et al., 2009; Koike et al., 2011a) and 24 h (Koike et al., 2011b) following a single injection of ketamine.

The most effective dose in the TST was 30 mg/kg. ICR mice were particularly sensitive to ketamine and continued to exhibit decreased immobility 72 h after treatment (Koike et al., 2011b). Furthermore, a lower dose of ketamine (10 mg/kg) was effective in reducing TST immobility increased by chronic mild stress (CMS) 48 h after ketamine injection (Ma et al., 2013). In contrast, two studies indicated that the acute reduction in immobility by high dose ketamine (50 and 160 mg/kg) was not maintained 1 week following treatment in mice (Popik et al., 2008; Bechtholt-Gompf et al., 2011). These data suggest that the TST is most valuable in the assessment of the more immediate antidepressant effects of ketamine. However, exposure to stress could increase the sensitivity to ketamine in the TST. To date there are no studies that have investigated whether the TST is sensitive to a chronic dosing regimen of ketamine.

### Novelty suppressed feeding (NSF)

Exposure to a novel environment produces an anxiety-like phenotype in rodents known as hyponeophagia. In the NSF and novelty-induced hypophagia (NIH) tests, the latency to feed is increased and the amount of food consumption is reduced in a novel environment. These tests, based on a similar principle, differ in methodology; NSF requires acute food deprivation 24 h prior to testing whereas the NIH utilizes an 8–10-day training period without deprivation. These tests have considerable face validity, although interpretation of results with the NSF may be limited by the use of food deprivation. Hyponeophagia is one of the few anxiety-related tests that are reliably attenuated following chronic, but not acute, administration of antidepressant drugs (Bodnoff et al., 1988; Dulawa and Hen, 2005). In contrast, ketamine reduced the latency to eat within hours of treatment. The effective dose range for ketamine in this task varied across studies: 30 min and 24 h following 5–10 mg/kg (Li et al., 2010; Carrier and Kabbaj, 2013) and 30 mg/kg (Iijima et al., 2012), but all tests resulted in a significant reduction in the latency to feed in the novel environment. Moreover, ketamine (10 mg/kg) successfully reduced the latency to eat in the NIH 1 h post-injection (Burgdorf et al., 2013). More protracted effects of acute ketamine treatment (3 mg/kg) were observed 48 h following treatment in mice exposed to chronic stress, although ketamine did not reduce feeding latency in stress naïve mice in this study (Autry et al., 2011).

Overall, these data suggest that hyponeophagia is highly sensitive to a single dose of ketamine, although additional parameters of these tests remain to be examined more systematically. The fact that ketamine produced anxiolytic effects rapidly whereas conventional antidepressants require chronic treatment for weeks agrees with a more rapid onset of clinical effects. As TRD patients exhibit increased comorbid anxiety compared to treatment responsive MDD patients, the usefulness of assessing ketamine in anxiety tests should not be overlooked.

### Sucrose preference test (SPT)

Sucrose consumption is widely accepted as a measure of anhedonia in rodents and has significant face validity in terms of its sensitivity to chronic stress and antidepressant treatment. Repeated administration of ketamine (7 days) reversed the decrease in sucrose consumption in rats exposed to chronic stress. Although it should be noted that this dosing regimen with ketamine also increased sweet food consumption in both stressed and non-stressed rats (Garcia et al., 2009). Furthermore, administration of a low dose of ketamine (0.5 mg/kg) for 10 days significantly increased sucrose consumption in WKY rats (Akinfiresoye and Tizabi, 2013). Marked increases in sucrose consumption in rats persisted at 1, 3, 5, and 7 days after a single treatment with ketamine (10 mg/kg) (Li et al., 2011), indicating significant protracted effects of ketamine on this behavior.

Decreases in sucrose consumption induced by exposure to LPS (Walker et al., 2013) and CMS (Ma et al., 2013) were reversed following a single ketamine treatment. Protracted effects of acute ketamine treatment were evident in CMS exposed mice tested at 4, 6, and 8 days after a single ketamine treatment (Ma et al., 2013). In contrast, the consumption of sugar pellets in CMS exposed rats was not altered by ketamine treatment (Rezin et al., 2009), although this particular test is not directly comparable to the traditional SPT. It should be noted that there is a lack of consensus on the most appropriate SPT protocol to model an anhedonic state in rats. Nevertheless, these data support the use of the SPT as a sensitive screening test for rapid-acting antidepressant-like drugs such as ketamine.

### Elevated plus maze (EPM)

The EPM is frequently used to measure anxiety behavior in rodents (Bourin, 1997; Rodgers et al., 1997) and has strong predictive validity for screening anxiolytics. However, it is generally not sensitive to antidepressant treatments. Ketamine induced an anxiolytic phenotype in rats during exposure to the EPM 30 min after a single ketamine injection (Engin et al., 2009). A similar effect was observed in mice 1 and 2 h following treatment (Hayase et al., 2006). These studies indicate that the EPM was not sensitive to low doses of ketamine; only higher doses (30 mg/kg) induced a significant anxiolytic effect. Moreover, lower doses of ketamine did not induce an anxiolytic response in the EPM in stress naïve mice (Autry et al., 2011). The lack of effect of low doses of ketamine is also characteristic of the TST. Parise and colleagues described significant anxiolytic effects in the EPM in rats 2 months after the completion of a 15-day dosing regimen of 20 mg/kg per day during adolescence (Parise et al., 2013). Although the presence of drug effects after such a long interval could indicate sensitivity to the protracted effects of ketamine, developmental factors may have played a greater role. At present the EPM can only be proposed as a tool for assessing the more immediate anxiolytic effects of ketamine.

### Locomotor activity

Antidepressant-like effects of ketamine are usually evaluated in conjunction with spontaneous activity, because increased motor activity can produce false positive effects in the aforementioned behavioral tasks. Ketamine produces significant hyperactivity immediately following injection; 10 min post i.p. injection of low dose ketamine (5–15 mg/kg), rats displayed hyperactivity in spontaneous activity (da Silva et al., 2010). In addition, repeated administration of ketamine (50 mg/kg) sensitized rats to its hyperactive effects (Popik et al., 2008).

However, most studies have reported either no change or a reduction of locomotor activity after ketamine. A reduction of open field behavior was produced by ketamine in rats at 30 min post 50 mg/kg (Engin et al., 2009) and 1 h post 10 and 25 mg/kg (Gigliucci et al., 2013). In addition, a single injection of ketamine did not alter locomotor activity beyond 30 min post-injection in rats (Reus et al., 2011; Tizabi et al., 2012; Yang et al., 2012; Akinfiresoye and Tizabi, 2013) or in mice (Lindholm et al., 2012). At 24 h post-injection, there was no effect on locomotor activity in mice by ketamine or by the NMDA antagonists CPP and MK-801 (Autry et al., 2011).

Furthermore, chronic administration of low dose ketamine did not affect spontaneous activity in adult rats (Garcia et al., 2008b; Ma et al., 2013). Interestingly, it was shown recently that hyperactivity was displayed in adolescent but not adult rats following chronic ketamine administration (Parise et al., 2013). Many of the experiments assessed in this review did not measure the effects of ketamine on locomotor activity at the dose and time point used. However, taken together, the data suggest it is important practice to assess changes in activity measures post-treatment to identify and eliminate the involvement of any potential locomotor effect in the behavioral responses to ketamine.

### Learned helplessness (LH)

The LH model of depression produces escape deficits in rodents exposed to unpredictable and uncontrollable stress (Seligman et al., 1980). LH is a popular model of depression as it has good face validity and induces a number of endophenotypes that can be measured in other behavioral tasks, including the FST and NSF. Repeated treatment with antidepressants reversed the coping behavior deficits in rats and mice (Shanks and Anisman, 1988; Caldarone et al., 2000). A single administration of ketamine (10 mg/kg) has been reported to reverse the deficits in coping behavior induced by learned helplessness 30–60 min (Beurel et al., 2011; Koike et al., 2011a) and 24 h after treatment (Maeng et al., 2008; Li et al., 2010). Furthermore, ketamine is effective in producing antidepressant-like effects in the LH in CMS-treated mice at even a lower dose (3 mg/kg) (Autry et al., 2011). Currently, there is no information regarding the protracted effects of ketamine in LH.

### Chronic mild stress (CMS)

Exposure to the CMS model induces depressive behavior in rodents following the presentation of a series of stressors in an unpredictable sequence over a prolonged period of time. CMS produces a number of behavioral changes in rodents thought to resemble features of depressed patients, such as anhedonia or loss of grooming (Willner, 1997, 2005). CMS satisfies most of the criteria of validity for an animal model of depression; it is etiologically relevant with good design, resulting in similar pathological alterations observed in humans that are sensitive to chronic antidepressant treatment. The behavioral and molecular changes induced by CMS are reversed by treatment with antidepressant drugs, but only after administration for several weeks. In contrast, ketamine reversed the behavioral and physiological alterations induced by CMS in rats following acute administration and the effects were maintained following chronic treatment. Acute and chronic treatment with ketamine reversed the increase in adrenal gland weight, promoted regain of body weight, and normalized circulating corticosterone and ACTH levels (Garcia et al., 2009). Physiological alterations induced by CMS were reversed by acute ketamine treatment in a similar study but failed to reverse CMS-induced anhedonia in the SPT (Rezin et al., 2009). In addition, CMS-exposed adolescent rats exhibited decreased immobility, increased sucrose consumption and latency to feed immediately following acute ketamine treatment (Parise et al., 2013).

Because the CMS is accepted as a rodent model of depression, CMS is an ideal paradigm with which to screen the antidepressant-like effects of novel therapeutics like ketamine. Reversal of CMS-induced depressive-like phenotypes measured using the mouse FST, NSF, and SPT has been reported by ketamine in the absence of any drug effect in stress naïve mice (Autry et al., 2011). Furthermore, the effect of ketamine in the NSF test was observed to persist in CMS mice 48 h post-injection. In line with these findings, two similar studies have indicated an increased sensitivity of CMS-exposed mice to ketamine (Li et al., 2011; Ma et al., 2013). Taken together, the CMS data is the most consistent and possibly the most valid method of examining the antidepressant-like effects of ketamine in preclinical studies.

## Ketamine—molecular mechanisms of action

In order to develop novel and more effective antidepressants, the molecular mechanisms underlying the protracted behavioral improvement associated with ketamine treatment need to be understood fully. The majority of this information has been garnered from preclinical animal studies and the principle findings are detailed in the following section.

### NMDA and AMPA receptors

Currently the hypothesis for ketamine's mechanism of action focuses on a cascade of neurochemical events that are initiated shortly after administration of ketamine. The events then persist in a protracted manner for days following its metabolism and elimination.

Reductions in neurogenesis and synaptic plasticity play a key role in the pathophysiology of MDD. Synaptic plasticity refers to the dynamic capability of synapses to form and retract processes, thereby modifying synaptic strength and communication. The most well studied mechanisms mediating changes in plasticity are long-term potentiation (LTP) and long-term depression (LTD). These processes involve significant alterations in pre and post-synaptic scaffolding proteins and glutamate receptors, primarily the glutamatergic receptor, α-amino-3-hydroxy-5-methyl-4-isoxazole propionic acid (AMPA). The AMPA receptor containing the subunits GluR1, GluR4, and GluR2 are involved in LTP, whereas GluR2, GluR3, and GluR4 are required for the AMPA receptor internalization needed to facilitate LTD (Kessels and Malinow, 2009). N-methyl-d-aspartate (NMDA) receptors at excitatory synapses are also subject to trafficking and significantly decrease in synaptic density during LTD (Peng et al., 2010). In the pyramidal cells of the hippocampus, LTP and LTD bidirectionally regulate dendritic spine growth and retraction, whereas AMPA expression is positively related to the size of the spine head. These dynamic processes are stabilized by concurrent alterations in the expression of synaptic proteins and signaling pathways.

Ketamine blocks NMDA receptors (NMDARs) at concentrations of 2–50 μm. The subsequent suppression of tonic glutamate input to GABAergic interneurons, results in disinhibition of glutamate signaling. This disinhibition and increase in glutamate neurotransmission is mediated by a decrease in GABAergic inhibitory feedback of the pyramidal neurons in layer V of the PFC, a region widely implicated in the development of psychiatric disorders (Homayoun and Moghaddam, 2007). Interestingly, post-mortem studies report reductions in pyramidal cells and GABAergic interneurons in the PFC of depressed individuals (Choudary et al., 2005; Rajkowska et al., 2007). Increases in glutamate will activate ionotropic AMPARs resulting in Na^2+^ influx and subsequent membrane depolarization, induction of signaling cascades and protein synthesis. Certain AMPARs that lack the GluR2 subunit actually result in Ca^2+^ influx (Kessels and Malinow, 2009). Upregulation of AMPA receptor expression following ketamine administration mediates the increased sensitivity to glutamate. It has been suggested that this increased sensitivity or “synaptic scaling” is necessary to maintain stability in synaptic plasticity and increased protein synthesis in the presence of chronic NMDAR blockade (Kavalali and Monteggia, 2012).

Pharmacological inhibition of ketamine's behavioral effects has been achieved using the AMPA receptor antagonist, 2, 3-dihdroxyl-6-nitro-7-sulfamoyl-benzo(f)quinoxaline-2, 3-dione (NBQX), reversing the antidepressant effects of ketamine in the LH paradigm (Maeng et al., 2008; Koike et al., 2011b). Furthermore, co-administration of AMPAR antagonists blocked the effects of ketamine in the FST (Autry et al., 2011). As AMPARs have a clear role in mediating ketamine's effects, a recent study showed the antidepressant-like effect of AMPA administration in the depressive-like WKY rats (Akinfiresoye and Tizabi, 2013). This data indicate that AMPA receptors and indeed the AMPA/NMDA ratio is an important consideration and target in the development of potential therapeutics.

### mTOR signaling

**Figure 1:**
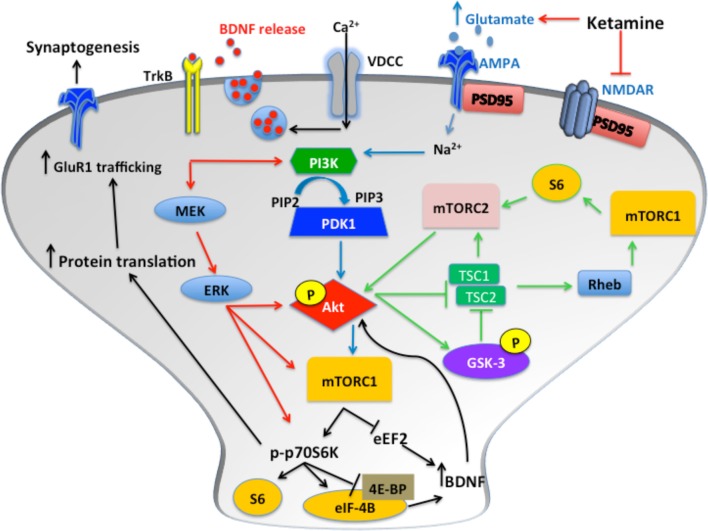
**Following blockade of NMDARs, phosphorylation of Akt activates mTOR complex 1 (mTORC1), which results in increased p70S6K phosphorylation and increased protein translation via inhibition of 4E-BP and release of eIF-4B**. Glutamate binds AMPARs, which induces depolarization of the membrane, enabling Ca^2+^ influx through VDCCs. This results in BDNF release from synaptic vesicles. The subsequent binding of TrkB receptors induces ERK and Akt signaling. These pathways all converge to increase synaptic protein translation and receptor trafficking to the cell membrane. Additionally, activation of mTORC2 by S6, and inhibition of GSK-3, induces mTORC1 activation via increased Akt phosphorylation. Furthermore, mTORC2 activation induces protein kinase C (PKC) signaling transduction, which regulates actin and other cytoskeletal proteins.

Data suggests that the protracted antidepressant-like effects of ketamine are mediated by molecular alterations to the signaling pathway for the mammalian target of rapamycin (mTOR) (see Figure 1), a serine/threonine kinase and key component of the insulin-signaling pathway (Li et al., 2010). Two functional mTOR complexes regulate the initiation of protein translation in mammalian cells, mTOR complex 1 (mTORC1) and mTOR complex 2 (mTORC2) (Rosner and Hengstschlager, 2011). A recent post-mortem study implicated decreases in cortical mTOR-signaling kinases in the pathophysiology of MDD (Jernigan et al., 2011). Additionally, rats exposed to CMS exhibit significant reductions in the phosphorylation of several kinases in the mTOR pathway in the amygdala of stressed rats (Chandran et al., 2013).

There is an inverted U-shape associated with ketamine-induced mTOR activation, with higher doses having no effect. In rodents, ketamine administration induced mTOR signaling approximately 30 min after injection. Li and colleagues elucidated some core features of ketamine's mechanism of action, primarily focusing on the alterations in mTOR dependent synapse formation in the PFC of rats (Li et al., 2010). In addition, they reported increased phosphorylation of mTOR, p70 KD ribosomal protein S6 kinase (p70S6K) and eukaryotic initiation factor 4E binding protein 1 (4E-BP1). P70S6K is required to inhibit suppression of eEF2, which prevents protein translation. Simultaneously, the phosphorylation of 4E-BP1 results in the release of eukaryotic translation initiation factor 4E (eIF-4E), thereby triggering the initiation of translation of synaptic proteins. These changes were accompanied by antidepressant-like behavior in the FST and NSF test (Li et al., 2010).

mTOR is ubiquitously expressed and has been found to localize in the cytoplasm of dendrites, where it can initiate the translation of synaptic proteins essential for the induction of LTP (Duman et al., 2012). PSD95, GluR1 and synapsin are upregulated approximately 2 h post-ketamine; this increase is observed for up to 72 h. Similarly, upregulation of Arc, a cytoskeletal protein is observed approximately 1 h post-injection and sustained for up to 6 h (Li et al., 2010). Arc is linked to the induction of early and late phase LTP and memory formation (Panja et al., 2009). A recent study confirmed that ketamine and MK-801 induced increases in immediate early genes, such as Arc, C-fos and Homer1a. Homer1a/Homer1b/PSD-95 signaling is implicated in glutamate induced synaptic plasticity (de Bartolomeis et al., 2013) and may be an interesting marker of plasticity for ketamine-like compounds. Similarly, reductions in the expression of eukaryotic elongation factor 2 (eEF2) is consistently observed in rodents following ketamine administration both in the PFC (Carrier and Kabbaj, 2013) and hippocampus (Autry et al., 2011). Interestingly, females are more sensitive to the behavioral effects of low dose ketamine compared to males; however, females do not exhibit decreases in eEF2 (Carrier and Kabbaj, 2013). Nevertheless, phosphorylation and inhibition of eEF2 may be a useful marker for rapid antidepressants, as increased phosphorylation of eEF2 in the PFC is also reported following chronic fluoxetine treatment in rats (Dagestad et al., 2006).

Pharmacological modulation of different components of the mTOR-signaling pathway (Figure 1) has been used to investigate mechanisms underlying the acute and protracted behavioral actions of ketamine. Inhibition of Akt, following blockade of phosphatidylinositol-3-kinase (PI3K) by LY294002, and inhibition of ERK using U0126, prevented ketamine reversal of CMS-induced deficits (Li et al., 2010). The Trk/B inhibitor K252a blocked the effects of ketamine in the TST and the NSF when tested 24 h, but not at 1 h (Koike et al., 2013b). The rapamycin-FKBP12 complex inhibits mTOR signaling when directly bound to mTORC1 (Hoeffer and Klann, 2010). Rapamycin pretreatment inhibited both the molecular and behavioral effects of ketamine on FST, NSF and the LH 24 h post-injection (Li et al., 2010). Furthermore, rapamycin administration did not inhibit the effects of ketamine in the NSF test at 30 min post-injection, but ketamine's effects were completely blocked at 24 h post-injection (Iijima et al., 2012). Thus, it appears that mTOR signaling is clearly associated with the protracted behavioral effects of ketamine measured 24 h later or longer, but other mechanisms may be involved in the immediate effects of ketamine, such as increased AMPAR activation. It is of interest to note that other antidepressants, including 5-HT_2C_ receptor antagonists, citalopram and electro-convulsive seizures (ECS, the equivalent to ECT in rodents) all increase mTORC1 levels (Elfving et al., 2013; Opal et al., 2013). However, the SSRI, sertraline, and the TCA, imipramine, actually have anti-proliferative effects that are mediated by inhibition of mTOR (Lin et al., 2010; Jeon et al., 2011). Furthermore, there is evidence that suggests rapamycin administration alone and the subsequent inhibition of mTOR signaling is capable of inducing antidepressant-like effects in the rat FST (Cleary et al., 2008). Moreover, the effects of long-term modulation of mTOR have yet to be assessed. These data indicate the role of mTOR signaling may be more complex than originally anticipated.

Other drugs have been used to identify neural mechanisms that might account for the antidepressant-like behavioral effects of ketamine. NMDA receptor blockade using MK-801 or CPP reduced immobility in the FST for up to 3 and 24 h, respectively, but neither compound reproduced the protracted effects of ketamine at longer intervals (Autry et al., 2011). The NR2B antagonist RO-25-6981 was suggested to induce mTOR signaling, resulting in similar molecular and behavioral effects as those observed following ketamine administration (Maeng et al., 2008; Li et al., 2010). In addition, the mGlu2/3 receptor antagonists LY341495 and MGS0039 decreased immobility time in the TST. NBQX had a limited effect on these antagonists, whereas rapamycin reversed the behavioral effects of these compounds at 24 h post-treatment, suggesting a role for mTOR signaling but not AMPA in mediating the antidepressant-like effects of mGluR2/3 antagonists (Koike et al., 2011a). The mGluR5 antagonist MPEP induced antidepressant-like effects in the NSF at 30 min and 24 h post-injection (Iijima et al., 2012). The effects at 24 h were blocked by rapamycin and the protein synthesis inhibitor anisomycin but not by the TrkB inhibitor K252a. In addition, the mGluR7 agonist AMN082 produced an antidepressant like effect in the TST 40 min post-injection which was reversed by NBQX pretreatment, suggesting that AMPA mediates the antidepressant effects of this compound (Bradley et al., 2012). Finally, the glycine functional partial agonist GLYX-13 produced an antidepressant-like effect in the FST, NIH and LH tests that extended for 24 h after injection, similar to the effects of ketamine (Burgdorf et al., 2013). These data suggest that when investigating the potential of novel compounds targeting glutamate, both mTOR and AMPA mediation should be assessed. Furthermore, it is important to choose an appropriate rodent strain in which to conduct these assays. For example, CD-1 mice are insensitive to modulation of the glutamatergic system and the subsequent antidepressant-like effects of AMNO82 and the mGluR 7 negative modulator MMPIP (O'Connor and Cryan, 2013).

### Brain-derived neurotrophic factor (BDNF)

Chronic administration of antidepressant drugs increases neurotrophins including BDNF (Duman and Monteggia, 2006). BDNF has high affinity for tyrosine kinase receptor B (TrkB), activating a number of signaling pathways that regulate neuronal growth and survival. This pathway also regulates the phosphorylation of cyclic-amp response element binding protein (CREB), which is integral to affective behavior, in addition to learning and memory (Autry and Monteggia, 2012). Post-mortem studies have reported reductions in BDNF and TrkB expression in the hippocampus and PFC of MDD patients and depressed suicides (Krishnan et al., 2007; Castren and Rantamaki, 2010; Yu and Chen, 2011). Rodent models of chronic stress and depression have recapitulated these region-specific changes of BDNF (Duman and Monteggia, 2006; Autry and Monteggia, 2012). At a behavioral level, BDNF administration reduces immobility in the FST (Shirayama et al., 2002; Hoshaw et al., 2005; Deltheil et al., 2008). Additionally, the over-expression of TrkB receptors leads to an antidepressant-like behavioral phenotype in mice (Koponen et al., 2004). BDNF deficient mice are depressive-like in some behavioral tests and fail to respond to conventional antidepressants in the CMS and FST compared to wild type mice (Saarelainen et al., 2003; Monteggia et al., 2007; Ibarguen-Vargas et al., 2009).

Activation of the mTOR pathway by ketamine enhances translation of BDNF in the hippocampus (Garcia et al., 2008a; Autry et al., 2011; Yang et al., 2012). The inhibition of eEF2 and subsequent increase in BDNF translation is proposed to mediate the rapid antidepressant-like effects of ketamine (Monteggia et al., 2013). Equally ketamine is capable of inducing a rapid release of glutamate. Following NMDA receptor blockade, AMPAR activation results in calcium influx via L-type voltage gated calcium channels (VDCC) inducing the release of BDNF from synaptic vesicles (see Figure 1). Furthermore, BDNF regulates neuronal mTOR function via Akt and PI3K, creating a positive feedback loop of BDNF production following the activation of mTOR by ketamine (Hay and Sonenberg, 2004; Hoeffer and Klann, 2010).

A single nucleotide polymorphism Val66Met (rs6265) in the BDNF gene has been proposed as a potential impediment to the antidepressant response to ketamine in TRD patients. Val/Val carriers are more sensitive to the antidepressant-effects of ketamine compared to the Val/Met carriers (Laje et al., 2012). However, not all studies have reported a positive correlation of improvement in depressive symptoms with increased BDNF (Machado-Vieira et al., 2009; Rybakowski et al., 2013). It is worth noting that BDNF serum concentrations were significantly lower in bipolar patients that did not respond to ketamine treatment compared to responders at baseline (Rybakowski et al., 2013). Mice that possess this polymorphism did not respond to ketamine and displayed significant impairments in synaptogenesis (Lindholm et al., 2012; Liu et al., 2012). However, at higher doses, repeated dosing or continuous infusion of ketamine, BDNF levels were increased, although this increase was correlated with neurodegeneration and cognitive deficits (Ibla et al., 2009; Goulart et al., 2010). Similarly, humans who chronically abuse ketamine exhibit higher BDNF concentrations compared to healthy controls (Ricci et al., 2011).

As a downstream product of multiple signaling cascades induced by ketamine, the production of BDNF occurs rapidly and may underlie the protracted behavioral response to ketamine. Indeed, acute i.c.v infusion of both BDNF and insulin-like growth factor (IGF-1) are capable of mediating protracted antidepressant like effects in the FST lasting up to 6 days following the infusion (Hoshaw et al., 2008). These data not only indicate that alterations in BDNF levels are most likely involved in the protracted effects of ketamine, but also confirms that rapid and persistent increases in neurotrophins are useful markers of novel rapid-acting antidepressants.

### Glycogen synthase kinase-3 (GSK-3)

GSK-3 is a serine/threonine protein kinase and a major target for the mood stabilizer lithium (Klein and Melton, 1996; Stambolic et al., 1996). TRD patients are often given a period of antidepressant augmentation treatment with lithium when they fail to response to SSRIs alone (Carvalho et al., 2007; Bauer et al., 2010). Furthermore, studies have shown that GSK-3 is functionally regulated by serotonin modulation, primarily mediated by 5-HT_1A_ autoreceptors and via iPI3K/Akt signaling (Polter et al., 2012). GSK-3β^±^ heterozygous mice display significant reductions in immobility in the FST (O'Brien et al., 2004). Interestingly mice with a knock-in mutation of GSK-3, which prevents its phosphorylation, do not respond to ketamine treatment in the LH paradigm, suggesting that some of ketamine's potential therapeutic efficacy might be mediated following inhibition of this kinase (Beurel et al., 2011). Furthermore, combination of ketamine and the GSK-3 inhibitor, SB216763, significantly reduced immobility in the FST; at a molecular level, this combination of ketamine and SB216763 amplified the frequency of 5-HT and hypocretin-induced EPSCs and increased spine density in the mPFC. Conversely, it had been shown that ketamine has limited effects on GSK-3 expression in hippocampal synaptosomes (Muller et al., 2013). Moreover, a single dose of ketamine reversed the behavioral effects of CMS, but the GSK-3 inhibitor SB216763 had no effect on CMS-induced behavioral scores (Ma et al., 2013). Further preclinical studies are required to evaluate the role of GSK-3β in the antidepressant-like response to ketamine. A recent assessment of three depressed patients indicates a significant increase in phosphorylated GSK-3β in the plasma of ketamine-treated individuals over the 120-min assessment period (Yang et al., 2013a). Although the inhibition of GSK-3β modulates m-TOR signaling (Figure 1) and may potentially augment the effects of antidepressants such as ketamine, it is unclear whether GSK-3 directly mediates the effects of ketamine.

## Conclusion and future directions

The development of ketamine as a rapidly acting antidepressant drug has the potential to revolutionize clinical treatment. Nevertheless, the clinical use of ketamine for depression poses a number of challenges. Ketamine is an hallucinogenic drug subject to abuse and must be given in a controlled setting. The effects of ketamine are short-lasting and can only be sustained by its repeated treatment. A desirable research direction would be to develop other drugs with similar antidepressant effects that are devoid of ketamine's liabilities. However, progress in this area is constrained by uncertainty concerning the critical pharmacological mechanisms underlying the antidepressant effects of ketamine.

Animal models have the potential to translate the pharmacological effects of ketamine that are most critical for its clinical antidepressant effects. A substantial body of literature now indicates that ketamine produces antidepressant-like effects in preclinical tests for antidepressant activity and in animal models of depression. Acute ketamine produces immediate effects on many behavioral tests that are similar to antidepressants. However, the protracted effects of ketamine measured for days after a single administration are not produced by conventional antidepressants. They define a new paradigm for antidepressant drug discovery that is the best temporal correlate with ketamine's clinical activity. Inconsistent findings across laboratories may arise from a disparity in methodology used across studies. The most pertinent variables are that the efficacious dose is dependent on the behavioral task employed, conditions surrounding administration and the time of testing post-administration of ketamine. For example, evidence suggests that the effects of low and seemingly sub-efficacious doses of ketamine are more effective following stress exposure. Behavioral tests with high predictive validity for antidepressant-like effects, such as the FST, are sensitive to acute and chronic ketamine. They can be utilized in conjunction with other tests sensitive only to chronic antidepressant treatment, such as the NSF/SPT, to measure the protracted benefits that are unique to ketamine. Overall, combination of a stress or genetic model of depression/anxiety with behavioral assessment over a 1–2 week period post-treatment with low doses of ketamine will yield the most valid and useful information.

Among the many barriers to translation of ketamine's clinical antidepressant effects across species stand a number of key pharmacological factors. The route of administration of ketamine in preclinical models is by i.p. injection, whereas intravenous infusion is usually employed in clinical trials. Therefore, it may be beneficial for animal studies to employ intravenous infusion where practical. In addition, plasma levels of ketamine monitored in the first 2 h following administration can determine whether the dose/route of administration of ketamine produces comparable bioavailability across species. Given that the half-life of ketamine is short, differing levels of ketamine may account for some variation in the behavioral tests. However, ketamine is no longer present when protracted behavioral effects are measured days after administration. These protracted changes result from rapid and sustained molecular alterations induced following a single treatment with ketamine. In addition, the preservative benzethonium chloride (BCl) is universally used in ketamine preparations both for clinical and preclinical use. Although present in low concentrations, BCl can act synergistically with ketamine to inhibit muscarinic and α7-nicotinic acetylcholine receptors (Durieux and Nietgen, 1997; Coates and Flood, 2001). The extent to which the additive properties of BCl on ketamine-induced modulation of the cholinergic system may affect the antidepressant-like response to ketamine is unknown. In the present review, there was no systematic evidence that positive or negative findings were associated with the source of ketamine in the behavioral studies examined here (Tables 1, 2).

The mechanisms underlying ketamine's effects, the simultaneous blockade of NMDA receptors and activation of AMPA receptors, are integral for the induction of the antidepressant response. The long-term consequences of these molecular alterations are likely to mediate ketamine's protracted antidepressant-like effects mediated via increased synaptic plasticity, neuronal survival and maturation. These changes occur within hours of ketamine administration and occur in parallel with both the rapid and protracted behavioral effects in animal models of depression. The rapid modulation of mTOR, its downstream mediators, such as Akt and ERK, and BDNF represent markers of the molecular correlates of the antidepressant effects of ketamine and its ability to modify synaptic plasticity. Novel therapeutics for TRD are likely to modulate these markers in a similar temporal pattern to that of ketamine and can be used to identify better pharmaceutical agents to treat TRD.

### Conflict of interest statement

The authors declare that the research was conducted in the absence of any commercial or financial relationships that could be construed as a potential conflict of interest.
